# Metformin, Asian ethnicity and risk of prostate cancer in type 2 diabetes: a systematic review and meta-analysis

**DOI:** 10.1186/s12885-017-3934-9

**Published:** 2018-01-10

**Authors:** Christopher B. Chen, Maxim Eskin, Dean T. Eurich, Sumit R. Majumdar, Jeffrey A. Johnson

**Affiliations:** 1 School of Public Health, University of Alberta, Edmonton, Alberta Canada; 2 Department of Medicine, Faculty of Medicine & Dentistry, University of Alberta, Edmonton, Alberta Canada; 3 2-040 Li Ka Shing Centre for Health Research Innovation, University of Alberta, Edmonton, AB T6G 2E1 Canada

**Keywords:** Metformin, Ethnicity & prostate cancer

## Abstract

**Background:**

Metformin is associated with a reduced risk of some cancers but its effect on prostate cancer is unclear. Some studies suggest only Asians derive this benefit. Therefore, we undertook a systematic review with particular attention to ethnicity.

**Methods:**

Medline, Embase, Scopus, Web of Science, and EBM Reviews were searched from inception to 2015. Two reviewers identified and abstracted articles. Studies were pooled using random effects model and stratified by Western- vs Asian-based populations.

**Results:**

We identified 482 studies; 26 underwent full review. Of Western-based studies (*n* = 23), two were randomized trials and 21 were observational studies. All Asian-based studies (*n* = 3) were observational. There were 1,572,307 patients, 1,171,643 Western vs 400,664 Asian. Across all studies there was no association between metformin and prostate cancer (RR: 1.01, 95%CI: 0.86-1.18, I^2^: 97%), with similar findings in Western-based trials (RR: 1.38, 95%CI: 0.72-2.64 I^2^: 15%) and observational studies (RR: 1.03 95%CI: 0.94-1.13, I^2^: 88%). Asian-based studies suggested a non-significant reduction (RR: 0.75, 95%CI: 0.42-1.34, I^2^: 90%), although these results were highly influenced by one study of almost 400,000 patients (propensity-adjusted RR: 0.47 95%CI 0.45-0.49). Removing this influential study yielded an estimate more congruent with Western-based studies (RR: 0.98 95%CI:0.71-1.36, I^2^: 0%).

**Conclusion:**

There is likely no association between metformin and risk of prostate cancer, in either Western-based or Asian-based populations after removing a highly influential Asian-based study.

**Electronic supplementary material:**

The online version of this article (10.1186/s12885-017-3934-9) contains supplementary material, which is available to authorized users.

## Background

Patients with type 2 diabetes have an increased risk of many cancers including breast, colorectal and endometrial cancer, but a reduced risk of prostate cancer. There are likely many factors involved in the relationship between diabetes and cancer, but increased cancer risk may be secondary to hyperinsulinemia induced cellular proliferation, while hyperinsulinemia may also lead to reduced testosterone levels, yielding a lower prostate cancer risk [1].

Metformin is an inexpensive and well-tolerated first line treatment for patients with type 2 diabetes. Metformin has also been associated with a reduced risk of some cancers, including colorectal, liver and lung [2]. However, the mechanism of this reduction has yet to be elucidated. This may be due to a direct effect of metformin to activate AMPK, which in turn inhibits mTOR, causing a decrease in cellular proliferation, or indirectly through its ability to reduce hyperinsulinemia and the associated cellular proliferation [3]. Regardless of the mechanism of action, this has prompted optimism for metfofmin’s role in cancer prevention. However, because hyperinsulinemia and type 2 diabetes have been associated with low testosterone, a reduction in circulating insulin may lead to increases in testosterone levels and subsequent proliferation of neoplastic cells in the prostate [4].

Further complicating the issue is potential effect modification by ethnicity. Specifically, relative to non-Asian patients with type 2 diabetes, Asian patients appear to have an increased risk of prostate cancer [5, 6]. While the biologic reasoning behind these difference are still in question, the disparate risk of prostate cancer between non-Asian and Asian patients may suggest differential effects of metformin on prostate cancer risk. Previous studies have investigated the association between metformin and prostate cancer, but with conflicting results that have not accounted for ethnicity. Therefore, we undertook this systematic review to summarize the association between metformin and risk of prostate cancer in Western- and Asian-based populations with type 2 diabetes.

## Methods

### Overview

We undertook a systematic review of the literature up to August 2015 using a pre-specified research protocol. Because ethnicity was not often explicitly identified, we stratified all analyses by source country, identifying studies as either Western-based (studies using populations based in Europe and North America) or Asian-based (predominantly studies based in Taiwan, no other studies used populations from other Asian countries).

### Literature search

The databases Pubmed/Medline, Scopus, Evidence Based Medicine Reviews (which includes ACP Journal Club, Cochrane Central Register of Controlled Trials, Cochrane Database of Systematic Reviews, Cochrane Methodology Register, Database of Abstracts of Reviews of Effects, Health Technology Assessments and National Health Service Economic Evaluation Database), Web of Science and Embase were searched from inception until August 2015. We used the search terms metformin, diabetes and cancer. Grey literature such as clinicaltrials.gov and conference abstracts from the American Diabetes Association and the European Association for the Study of Diabetes were also searched.

Manuscript and abstract titles were reviewed and those potentially relevant to our objective were recorded. Two reviewers (CC and ME) then independently analyzed the abstracts and full texts of those recorded. We included observational studies or randomized controlled trials that investigated the incidence of prostate cancer in adult populations, and were comparing those currently exposed to metformin versus those who are not. Additionally, the reference lists were hand searched and experts contacted. Any conflicts regarding study inclusion were reconciled through discussion with the senior author (JAJ) who was the final arbiter of inclusion or exclusion if consensus could not be achieved.

### Data abstraction

Two reviewers (CC and ME) independently abstracted information regarding study design, data source, study region (i.e., Western-or Asian-based), exposure and comparator, number of patients in each exposure group, study period, length of follow up, covariates adjusted for, fully adjusted and crude odds ratios, risk ratios or hazard ratios and confidence intervals. If multiple risk estimates were available, the most completely adjusted value was taken as the primary risk estimate, but we also abstracted unadjusted comparisons where available. Any discrepancies were reconciled by consensus after referring to the original report. The risk of bias of each study was determined with the Newcastle-Ottawa scale for observational studies and the Cochrane Risk of Bias tool for randomized controlled trials [7, 8].

### Data analysis

For observational studies, the adjusted and unadjusted (where available) hazard ratios and confidence intervals were pooled using the random effects model and the inverse variance method. Because randomization balanced measured and unmeasured confounders, we pooled unadjusted risk estimates from the controlled trials using the Mantel-Haenszel method. Heterogeneity was assessed using the I^2^ parameter. We stratified our results by study type, study region (i.e., Western-or Asian-based) and risk of bias for observational studies (stratified by the median Newcastle-Ottawa score of 6). Publication bias was assessed with visual inspection of funnel plots. All analyses and calculations were completed in RevMan 5.3 (Copenhagen, Denmark).

## Results

Our initial literature search identified 501 titles of potential interest, once duplicates were removed. After initial review of abstracts and full texts, 22 studies were identified. Two hundred and two (42%) studies were excluded because they lacked metformin exposure and 277 (58%) were excluded because they did not study incident prostate cancer cases. An additional 6 articles were identified from their reference lists, yielding a total of 28 studies that were abstracted**,** including two randomized trials and 26 observational studies. There were 2 Western-based randomized trials, 3 Asian-based cohort studies, 1 Asian-based case-control study, 14 Western-based cohort studies and 8 Western-based case-control studies [9–33]. One study (Geraldine et al.) did not have sufficient information to be included in the pooled analyses and Tseng 2014 used a similar cohort to Tseng 2011, hence only Tseng 2014 was included, providing a total of 26 studies included in the quantitative analysis. There were a total of 1,572,307 patients, 1,171,643 Western vs 400,664 Asian. There was insufficient information to tabulate the crude total number of events. The median Newcastle-Ottawa score was 6.

**Fig. 1:**
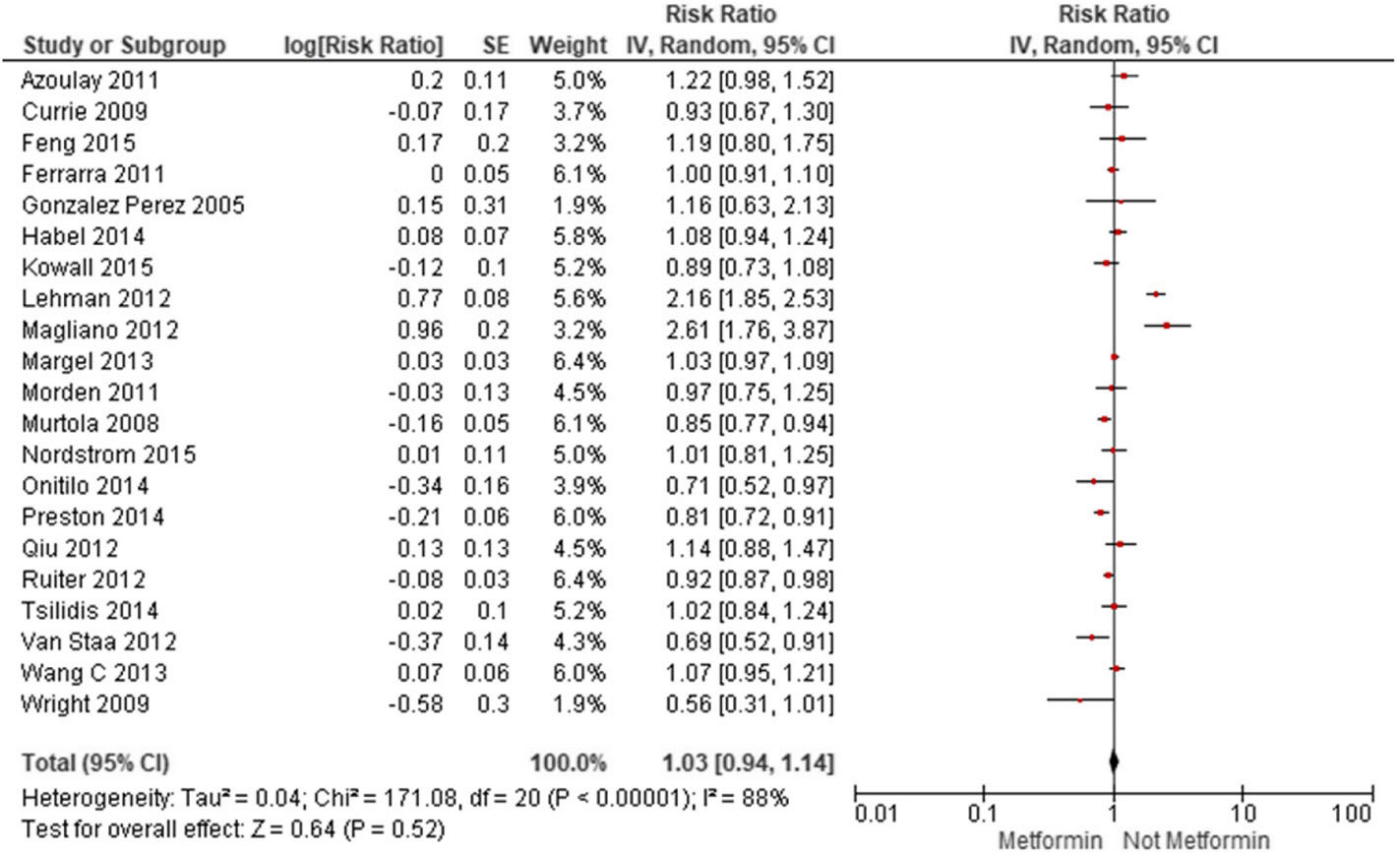
Current Metformin Use Vs. No Current Metformin Use in Western-Based Observational Studies

**Fig. 2:**
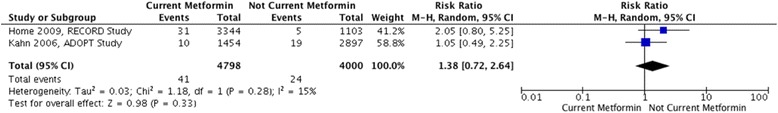
Current Metformin Use Vs. No Current Metformin Use in Western-Based Randomized Trials

**Fig. 3:**
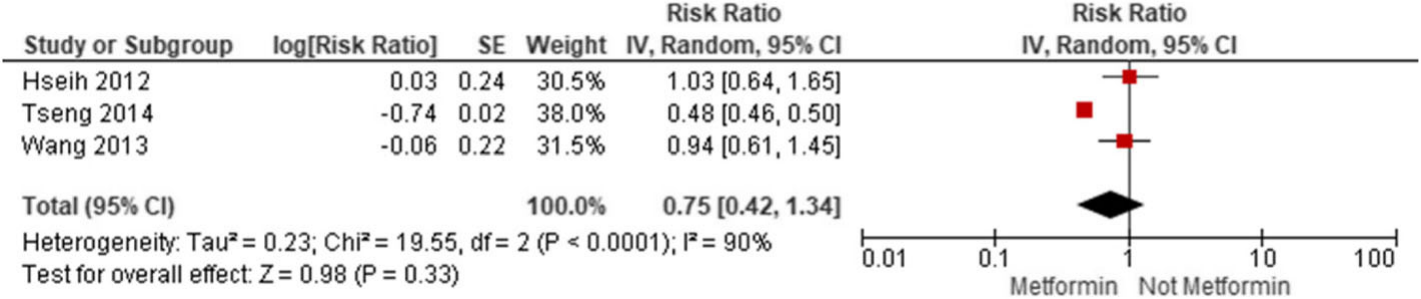
Current Metformin Use Vs. No Current Metformin Use in Asian-Based Observational Studies

There was no association between metformin use and prostate cancer observed among Western-based studies, whether observational (RR: 1.03; 0.94-1.13, I^2^: 88%) (Fig. 1) or trials (RR: 1.38; 0.72-2.64 I^2^: 15%) (Fig. 2). Asian-based observational studies suggested a non-significant reduction in prostate cancer (RR: 0.75; 0.42-1.34, I^2^: 90%) (Fig. 3), but these results were highly influenced by one single study of almost 400,000 patients, with a propensity-adjusted RR of 0.48 (95%CI: 0.45-0.50) [21]. Removing this one study yielded a much lower heterogeneity estimate more congruent with estimates of effect from Western-based studies (RR: 0.98; 0.71-1.36, I^2^: 0%). We did not identify any Asian-based clinical trials.

Stratification by study design and risk of bias provided similar results. The pooled risk estimates of adjusted data comparing current metformin use against no current metformin use in all cohort studies, Western-based and Asian-based cohort studies were: 1.01 (0.80, 1.28; I^2^: 98%), 1.07 (0.93, 1.23; I^2^: 91%) and 0.68 (0.32, 1.44; I^2^: [7]90%), respectively (Additional file 1, Additional file 2 and Additional file 3). In all case-control studies, Western-based and Asian-based case-control studies, the pooled risk estimates were: 0.95 (0.85, 1.07; I^2^: 73%), 0.96 (0.84, 1.08; I^2^: 76%) and 0.94 (0.61, 1.46; I^2^: not calculable), respectively (Additional file 4, Additional file 5 and Additional file 6). Similar risk estimates, with substantial heterogeneity, were found when observational studies were stratified by median score of 6 on the Newcastle-Ottawa Scale (Additional file 7, Additional file 8, Additional file 9, Additional file 10, Additional file 11, Additional file 12, Additional file 13 and Additional file 14).

The pooled risk ratio of crude data comparing current metformin use against no current metformin use in all observational studies was 0.83 (0.65,1.07) with an I^2^ of 98% (Additional file 15). The risk ratio for Western-based observational studies was 0.86 (0.69, 1.07) with an I^2^ of 95% and for Asian-based observational studies was 0.66 (0.43,1.03) with an I^2^ of 74%, which again, was heavily influenced by the larger Tseng 2014 study with an extreme HR (Additional file 16 and Additional file 17).

## Discussion

At an initial glance, our synthesis of the available evidence suggests no association between metformin exposure and prostate cancer risk, a finding that is congruent in observational studies whether Western-based or Asian-based. A lower risk of prostate cancer with metformin exposure was evident in Asian-based studies, although statistically insignificant, and heavily influenced by a single, large study from Taiwan with an extreme risk estimate [21]. Removing this study returns this association to the null, and removes substantial heterogeneity. Although based on its Newcastle-Ottawa score, this study was not more biased than other studies, had a high level of precision and had a large sample size. Thus it may inappropriate to exclude this study from the pooled estimates. In fact, this study may act as a reference point in the funnel plot that all other studies should be compared against. However, given its large sample size and subsequent influence on the pooled risk estimate, we felt it prudent to explore the effect of this study on the pooled risk estimate by excluding it from pooled risk estimate as a type of sensitivity analysis.

Only two clinical trials were identified thus insufficient data from a study design with a high level of methodological rigour are available. Furthermore, observational studies do not identify an association but contradict the study with the largest sample size. Moreover, the funnel plot contains points clustered around the null and does not resemble a funnel shape. This may indicate that there are unpublished or unidentified studies or that metformin exposure has disparate effects on disparate study characteristics such as age or utilization of metformin. Thus there is currently insufficient evidence to form strong conclusions regarding the association between ethnicity, metformin exposure and prostate cancer. However, the current evidence may suggest that there is no association between metformin exposure, ethnicity and prostate cancer incidence.

Previous systematic reviews on the topic have found similar results, with no statistically significant association between metformin exposure and prostate cancer risk. Gandini et al. associated metformin exposure with a 1.06 (0.80,1.41) relative risk and an I^2^ of 91% [2]. However they could only pool 12 studies at the time. Franciosi et al. achieved a similar pooled estimate from observational studies, 0.96 (0.87, 1.05) with an I^2^ of 60% but pooled certain studies more than once [34]. Noto et al. pooled 7 studies and found a risk estimate of 0.89 (0.66, 1.19) with an I^2^ of 66% [35]. Similarly, Soranna et al. found a pooled estimate of 0.92 (0.73, 1.17) with an I^2^ of 78% [36]. Wu et al. pooled 10 studies yielding an estimate of 0.92 (0.84, 1.03) with an I^2^ of 71% [37]. However, Yu et al. and Deng et al. found a slight statistically significant reductions in prostate cancer risk associated with metformin use, of 9% and 12%, respectively, although with substantial (50-75%) heterogenetity [38, 39]. Thus, our results agree with most previous systematic reviews, which were also limited by significant heterogeneity, while including more recent studies. Moreover, our specific focus on the stratification by ethnicity has specifically addressed one potential source of heterogeneity.

Despite some strengths, the review possesses some limitations. The first and most significant limitation is the lack of individual patient data, thus we were relegated to stratifying by ethnicity based on the origin of the database. These databases may contain patients of several ethnicities and any potential ensuing misclassification may have biased our results. Furthermore, despite stratification by ethnicity, study design and risk of bias, significant heterogeneity was observed. Because analysis using crude values yielded I^2^ ranging from 74% to 98%, the observed heterogeneity may not be solely due to disparate methods of statistical adjustment. Instead it may be a result of different patient populations or methodological heterogeneity. Regardless, pooling may not be the most accurate depiction of the association between metformin exposure and prostate cancer risk. Furthermore, pooling two Western-based clinical trials likely does not provide reliable results, thus we are limited to reporting the pooled estimate from Western-based clinical trials on a narrative basis. Similarly, other analyses in our supplemental materials only included one or two studies which would not provide reliable estimates and are only provided for illustrative purposes.

What may also be considered a major limitation is the inconsistent drug exposure definitions used in each study, which may have also contributed to the observed heterogeneity. While some studies defined metformin exposure using an ever/never definition or metformin use/no use definitions, other studies compared metformin use against sulfonylurea use or diet. Ideally, the association between metformin exposure and cancer risk would account for time-varying and accumulated drug exposure [40]. Van Staa et al., Azoulay et al., Preston et al. and Margel et al. evaluated the association between prostate cancer risk and cumulative metformin exposure [9, 18, 23, 26]. Among these studies, higher metformin doses were associated with increased, decreased and no association with any prostate cancer risk. Preston et al. did not associate higher doses of metformin exposure with any prostate cancer but with a reduced risk of localized prostate cancer [23]. On the contrary, Margel et al. found no association between cumulative metformin exposure and low- or high-grade prostate cancer [18]. This presents another potential factor in to our research questions, suggesting that definition, cumulative dose or duration of metformin exposure, as well as prostate cancer grade, may influence the association between metformin and prostate cancer risk.

Moreover, body mass index (BMI) has been associated with increased aggressive prostate cancer risk, which may be particularly important in our study [41]. Non-Asian-Americans with normal BMIs possess lower mean prostate specific antigen levels than Asian-Americans with normal BMIs while non-Asian-Americans who are overweight or obese have higher levels than overweight or obese Asian-Americans. Thus BMI introduces an additional variable that may affect the potential association between metformin exposure, ethnicity and prostate cancer incidence. Further, most studies lacked adjustment for other prognostic clinical variables such as family history of cancer. Unfortunately, these could not be adequately explored in our review because not all studies adjusted for BMI or other clinical covariates and individual level data was not available.

## Conclusion

There was insufficient evidence identified to form strong conclusions regarding the association between metformin exposure, prostate cancer and ethnicity. However, from the currently available evidence, we found no association between metformin and risk of prostate cancer, and this lack of association was present irrespective of ethnicity. While research with more robust methods and analysis such as a more accurate classification of ethnicity, consistent adjustment for BMI and more accurate definitions of metformin exposure (i.e., individual patient data) would be welcomed, our results may begin to temper the previous enthusiasm around the potential benefits of metformin on the risk of developing prostate cancer. However, because users and non-users of metformin do not have disparate risks of prostate cancer, it may be possible that metformin negates the reduced risk of prostate cancer between patients with and without diabetes.

## Additional files

Additional file 1: Current Metformin Use Vs. No Current Metformin Use in Western- and Asian-Based Cohort Studies. Comparison of metformin use in western and asian-based cohort studies. (DOCX 26 kb)

Additional file 2: Current Metformin Use Vs. No Current Metformin Use in Western-Based Cohort Studies. Comparison of metformin use in western-based cohort studies. (DOCX 25 kb)

Additional file 3: Current Metformin Use Vs. No Current Metformin Use in Asian-Based Cohort Studies. Comparison of metformin use in Asian-based cohort studies. (DOCX 17 kb)

Additional file 4: Current Metformin Use Vs. No Current Metformin Use in Western- and Asian-Based Case-Control Studies. Comparison of metformin use in Western- and Asian-based case-control studies. (DOCX 22 kb)

Additional file 5: Current Metformin Use Vs. No Current Metformin Use in Western-Based Case-Control Studies. Comparison of metformin use in Western-based case-control studies (DOCX 22 kb)

Additional file 6: Current Metformin Use Vs. No Current Metformin Use in Asian-Based Case-Control Studies. Comparison of metformin use in Asian-based case-control studies (DOCX 16 kb)

Additional file 7: Risk of Bias ≤6; Current Metformin Use Vs. No Current Metformin Use in Western- and Asian-Based Cohort Studies. Comparison of metformin use in Western- and Asian-based cohort studies with a Newcastle-Ottawa score ≤ 6. (DOCX 21 kb)

Additional file 8: Risk of Bias ≤6; Current Metformin Use Vs. No Current Metformin Use in Western-Based Cohort Studies. Comparison of metformin use in Western-based cohort studies with a Newcastle-Ottawa score ≤ 6. (DOCX 20 kb)

Additional file 9: Risk of Bias ≤6; Current Metformin Use Vs. No Current Metformin Use in Asian-Based Cohort Studies. Comparison of metformin use in Asian-based cohort studies with a Newcastle-Ottawa score ≤ 6. (DOCX 18 kb)

Additional file 10: Risk of Bias ≤6; Current Metformin Use Vs. No Current Metformin Use in Western- and Asian-Based Case-Control Studies. Comparison of metformin use in Western- and Asian-based case-control studies with a Newcastle-Ottawa score ≤ 6. (DOCX 20 kb)

Additional file 11: Risk of Bias ≤6; Current Metformin Use Vs. No Current Metformin Use in Western-Based Case-Controls Studies. Comparison of metformin use in Western-based case-control studies with a Newcastle-Ottawa score ≤ 6. (DOCX 19 kb)

Additional file 12: Risk of Bias ≤6; Current Metformin Use Vs. No Current Metformin Use in Asian Based Case-Control Studies. Comparison of metformin use in Asian-based case-control studies with a Newcastle-Ottawa score ≤ 6. (DOCX 17 kb)

Additional file 13: Risk of Bias >6; Current Metformin Use Vs. No Current Metformin Use in Western-Based Cohort Studies. Comparison of metformin use in Western-based cohort studies with a Newcastle-Ottawa score > 6. (DOCX 21 kb)

Additional file 14: Risk of Bias >6; Current Metformin Use Vs. No Current Metformin Use in Western Based Case Control Studies. Comparison of metformin use in Western-based case-control studies with a Newcastle-Ottawa score > 6. (DOCX 18 kb)

Additional file 15: Current Metformin Use Vs. No Current Metformin Use in Western- and Asian-Based Observational Studies. Comparison of metformin use in Western- and Asian-based observational studies. (DOCX 26 kb)

Additional file 16: Current Metformin Use Vs. No Current Metformin Use in Western- Based Observational Studies. Comparison of metformin use in Western-based observational studies. (DOCX 18 kb)

Additional file 17: Current Metformin Use Vs. No Current Metformin Use in Asian-Based Observational Studies. Comparison of metformin use in Asian-based observational studies. (DOCX 14 kb)

## Abbreviations

AMPK: AMP Activated Protein Kinase
BMI: Body Mass Index
mTorr: Mammalian Target of Rapamycin
RR: Risk Ratio
