# Supplementary figures and images for: Metformin, Asian ethnicity and risk of prostate cancer in type 2 diabetes: a systematic review and meta-analysis
Source: BMC Cancer. 2018 Jan 10;18:65. doi: 10.1186/s12885-017-3934-9 (PMC5763543; doi:10.1186/s12885-017-3934-9)

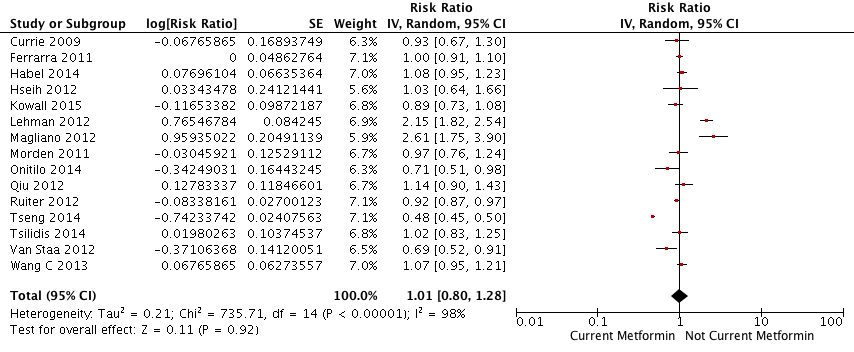

Supplement: Supplementary file 1 — Current Metformin Use Vs. No Current Metformin Use in Western- and Asian-Based Cohort Studies. Comparison of metformin use in western and asian-based cohort studies. (DOCX 26 kb) [file 12885_2017_3934_MOESM1_ESM.docx]

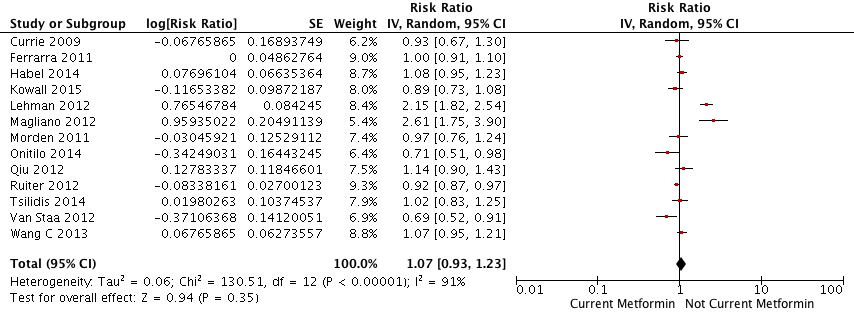

Supplement: Supplementary file 2 — Current Metformin Use Vs. No Current Metformin Use in Western-Based Cohort Studies. Comparison of metformin use in western-based cohort studies. (DOCX 25 kb) [file 12885_2017_3934_MOESM2_ESM.docx]

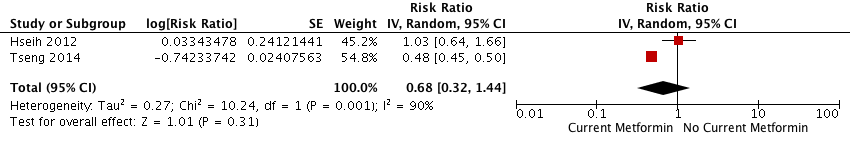

Supplement: Supplementary file 3 — Current Metformin Use Vs. No Current Metformin Use in Asian-Based Cohort Studies. Comparison of metformin use in Asian-based cohort studies. (DOCX 17 kb) [file 12885_2017_3934_MOESM3_ESM.docx]

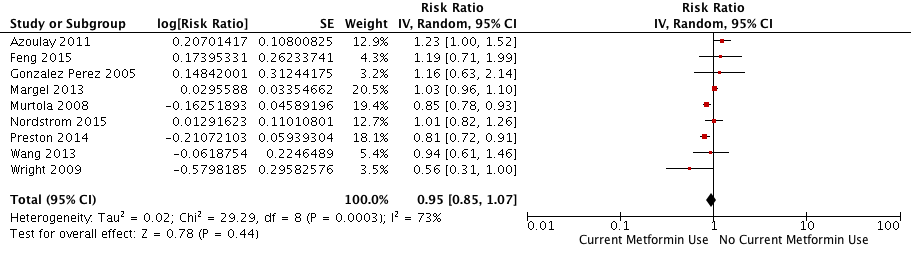

Supplement: Supplementary file 4 — Current Metformin Use Vs. No Current Metformin Use in Western- and Asian-Based Case-Control Studies. Comparison of metformin use in Western- and Asian-based case-control studies. (DOCX 22 kb) [file 12885_2017_3934_MOESM4_ESM.docx]

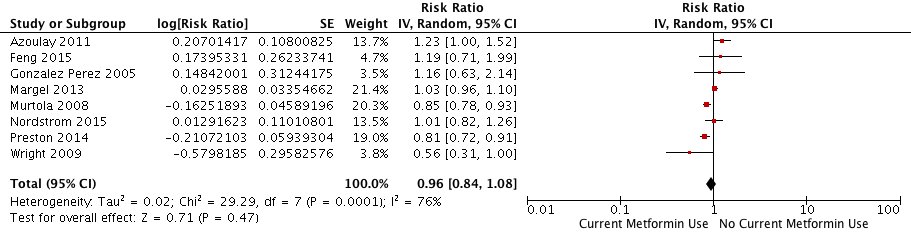

Supplement: Supplementary file 5 — Current Metformin Use Vs. No Current Metformin Use in Western-Based Case-Control Studies. Comparison of metformin use in Western-based case-control studies (DOCX 22 kb) [file 12885_2017_3934_MOESM5_ESM.docx]

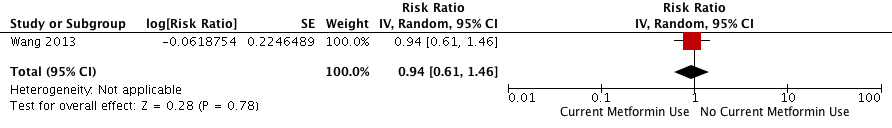

Supplement: Supplementary file 6 — Current Metformin Use Vs. No Current Metformin Use in Asian-Based Case-Control Studies. Comparison of metformin use in Asian-based case-control studies (DOCX 16 kb) [file 12885_2017_3934_MOESM6_ESM.docx]

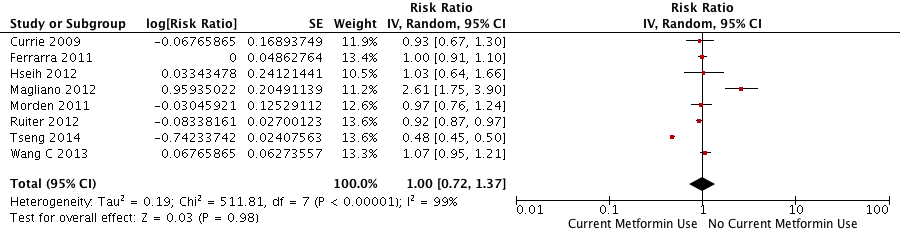

Supplement: Supplementary file 7 — Risk of Bias ≤6; Current Metformin Use Vs. No Current Metformin Use in Western- and Asian-Based Cohort Studies. Comparison of metformin use in Western- and Asian-based cohort studies with a Newcastle-Ottawa score ≤ 6. (DOCX 21 kb) [file 12885_2017_3934_MOESM7_ESM.docx]

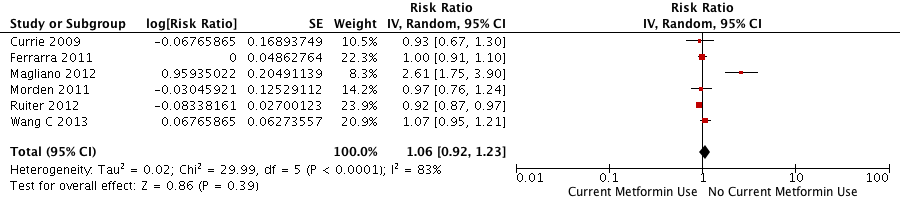

Supplement: Supplementary file 8 — Risk of Bias ≤6; Current Metformin Use Vs. No Current Metformin Use in Western-Based Cohort Studies. Comparison of metformin use in Western-based cohort studies with a Newcastle-Ottawa score ≤ 6. (DOCX 20 kb) [file 12885_2017_3934_MOESM8_ESM.docx]

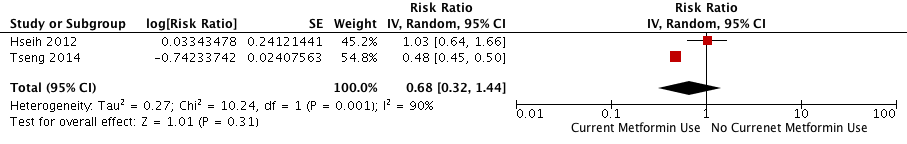

Supplement: Supplementary file 9 — Risk of Bias ≤6; Current Metformin Use Vs. No Current Metformin Use in Asian-Based Cohort Studies. Comparison of metformin use in Asian-based cohort studies with a Newcastle-Ottawa score ≤ 6. (DOCX 18 kb) [file 12885_2017_3934_MOESM9_ESM.docx]

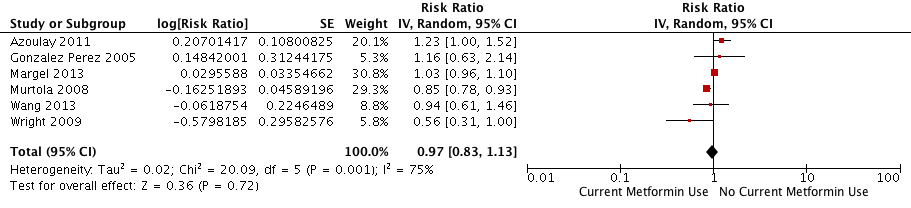

Supplement: Supplementary file 10 — Risk of Bias ≤6; Current Metformin Use Vs. No Current Metformin Use in Western- and Asian-Based Case-Control Studies. Comparison of metformin use in Western- and Asian-based case-control studies with a Newcastle-Ottawa score ≤ 6. (DOCX 20 kb) [file 12885_2017_3934_MOESM10_ESM.docx]

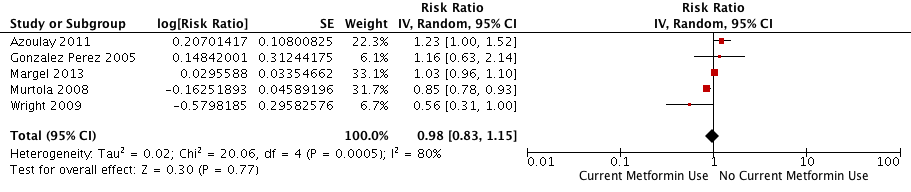

Supplement: Supplementary file 11 — Risk of Bias ≤6; Current Metformin Use Vs. No Current Metformin Use in Western-Based Case-Controls Studies. Comparison of metformin use in Western-based case-control studies with a Newcastle-Ottawa score ≤ 6. (DOCX 19 kb) [file 12885_2017_3934_MOESM11_ESM.docx]

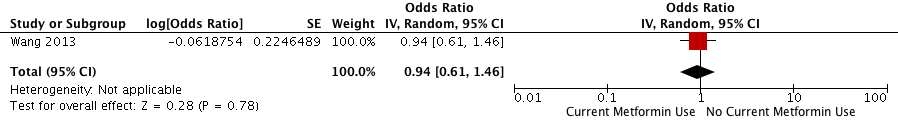

Supplement: Supplementary file 12 — Risk of Bias ≤6; Current Metformin Use Vs. No Current Metformin Use in Asian Based Case-Control Studies. Comparison of metformin use in Asian-based case-control studies with a Newcastle-Ottawa score ≤ 6. (DOCX 17 kb) [file 12885_2017_3934_MOESM12_ESM.docx]

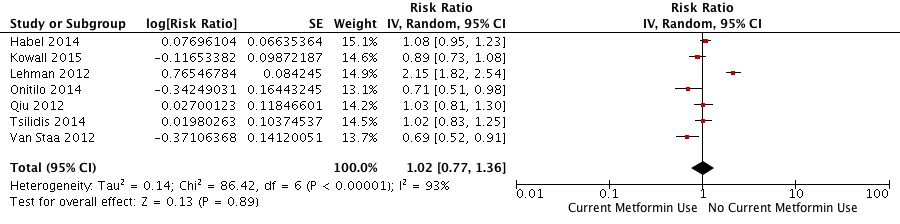

Supplement: Supplementary file 13 — Risk of Bias >6; Current Metformin Use Vs. No Current Metformin Use in Western-Based Cohort Studies. Comparison of metformin use in Western-based cohort studies with a Newcastle-Ottawa score > 6. (DOCX 21 kb) [file 12885_2017_3934_MOESM13_ESM.docx]

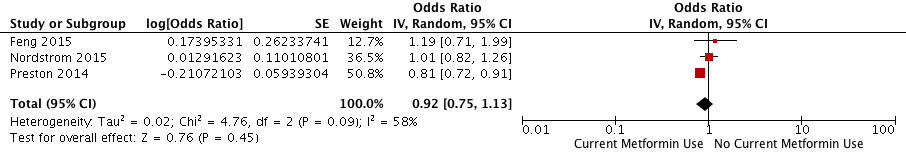

Supplement: Supplementary file 14 — Risk of Bias >6; Current Metformin Use Vs. No Current Metformin Use in Western Based Case Control Studies. Comparison of metformin use in Western-based case-control studies with a Newcastle-Ottawa score > 6. (DOCX 18 kb) [file 12885_2017_3934_MOESM14_ESM.docx]

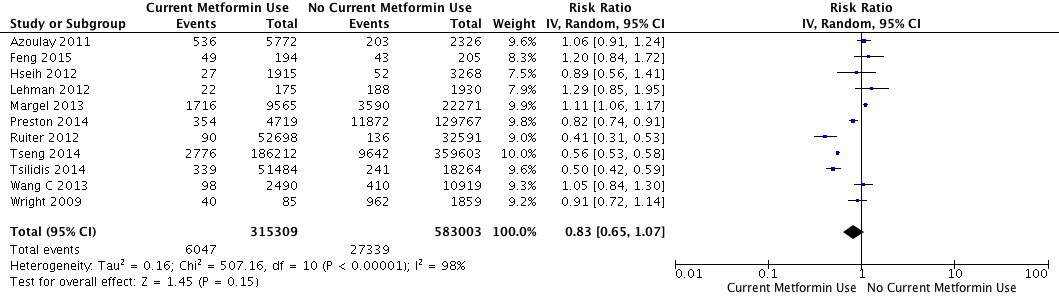

Supplement: Supplementary file 15 — Current Metformin Use Vs. No Current Metformin Use in Western- and Asian-Based Observational Studies. Comparison of metformin use in Western- and Asian-based observational studies. (DOCX 26 kb) [file 12885_2017_3934_MOESM15_ESM.docx]

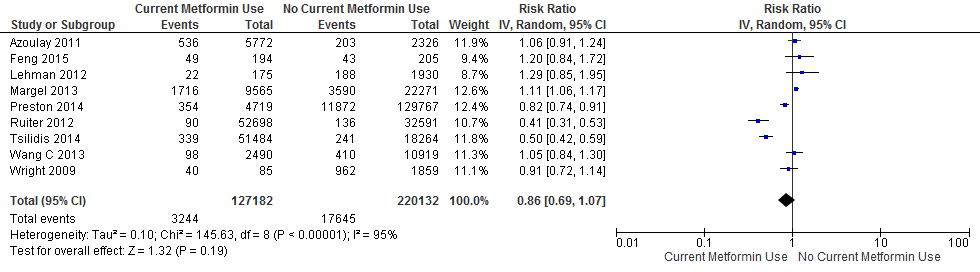

Supplement: Supplementary file 16 — Current Metformin Use Vs. No Current Metformin Use in Western- Based Observational Studies. Comparison of metformin use in Western-based observational studies. (DOCX 18 kb) [file 12885_2017_3934_MOESM16_ESM.docx]

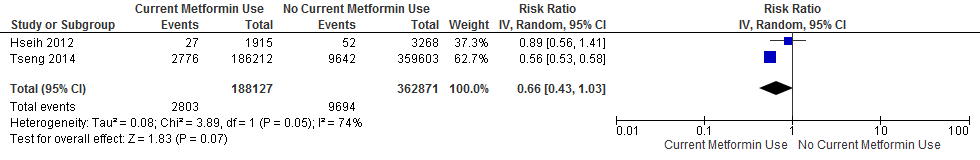

Supplement: Supplementary file 17 — Current Metformin Use Vs. No Current Metformin Use in Asian-Based Observational Studies. Comparison of metformin use in Asian-based observational studies. (DOCX 14 kb) [file 12885_2017_3934_MOESM17_ESM.docx]
